# Abdominal obesity as assessed by anthropometric measures associates with urinary incontinence in females: findings from the National Health and Nutrition Examination Survey 2005–2018

**DOI:** 10.1186/s12905-024-03059-2

**Published:** 2024-04-02

**Authors:** Ting Long, Bohuai Cheng, Ke Zhang

**Affiliations:** 1 Department of Pelvic Floor, Hunan Provincial Maternal and Child Health Care Hospital, Changsha, 410007 China; 2 Department of Otorhinolaryngology Head and Neck Surgery, The Sixth Affiliated Hospital, Sun Yat-sen University, Guangzhou, 510655 China

**Keywords:** Body roundness index, Conicity index, Waist-to-height ratio, Abdominal obesity, Urinary incontinence

## Abstract

**Background:**

Urinary incontinence (UI) is significantly link to abdominal obesity. This study aimed to assess the association between anthropometric indices of abdominal obesity, including body roundness index (BRI), conicity index (CI), and waist-to-height ratio (WHtR), and UI risk in adult females.

**Methods:**

We analyzed data from 10, 317 adult females in the National Health and Nutrition Examination Survey (NHANES) database (2005–2018). Weighted multivariable-adjusted regression analysis was conducted to determine the odds ratio (OR) and 95% confidence intervals (CI) for the association between BRI, CI, WHtR, and UI. Stratified analyses revealed the association based on the population type. Receiver operating characteristic curve (ROC) analyses were used to assess the predictive value of UI.

**Results:**

All indices of abdominal obesity investigated were positively and independently associated with the prevalence and severity of three types of UI. After adjusting for all relevant confounding variables, a significantly positive association between BRI and the prevalence of UI were observed (OR quartile 4 vs. quartile 1: urge UI (UUI): 1.93, 95% CI 1.61–2.30; stress UI (SUI): 2.29, 95% CI 1.94–2.70; mixed UI (MUI): 2.26, 95% CI 1.82–2.82; all *P* < 0.0001, *P* for trend < 0.0001, respectively), as well as WHtR and CI, which particularly prominent for female in premenopausal. Moreover, a one-unit increment of BRI was significantly associated with an increased severity index of UUI (β: 0.06, 95% CI 0.04–0.09, *P* < 0.0001), SUI (β: 0.10, 95% CI 0.07–0.13, *P* < 0.0001) and MUI (β: 0.07, 95% CI 0.04–0.10, *P* < 0.0001), which this trend was also observed in each subtype of UI for WHtR and CI. Furthermore, the ROC analysis demonstrated a higher diagnostic efficacy of BRI and WHtR compared with BMI in discriminating UI with an AUC of 0.600 for SUI, 0.617 for UUI, and 0.622 for MUI (all *P* < 0.05).

**Conclusions:**

An increased BRI, CI, and WHtR are significantly associated with higher prevalence and severity of UI in females.

**Supplementary Information:**

The online version contains supplementary material available at 10.1186/s12905-024-03059-2.

## Introduction

Urinary incontinence (UI) is a prevalent condition that characterizes the involuntary loss of bladder control. It more commonly affects female and presents with various types, including urge UI (UUI), stress UI (SUI), and mixed UI (MUI), which is a combination of both SUI and UUI [1]. Despite its treatable nature, patients with UI often suffer from embarrassment and psychological distress, hindering them from seeking medical attention and exacerbating their symptoms further [2]. Owing to factors such as pregnancy, obesity, chronic disease, and increasing age, UI has a high prevalence with 53% among females, and 10% of female had UUI, 26% had SUI and 16% had MUI [3]. In addition, UI significantly affects the quality of life, results in enormous physical, social, and psychological adverse consequences, thereby creating a critical public health burden [4].

Obesity, especially abdominal obesity, is a significant risk factor for the development of UI [5]. Studies suggested that increased intra-abdominal pressure due to abdominal obesity places additional stress on the pelvic floor, leading to the development of SUI [6]. Similarly, UUI may also be induced after gaining weight [7]. Specifically, intra-abdominal pressure increases with abdominal obesity, thereby weakening pelvic muscles and pelvic nerve innervation [6]. Another potential physiological mechanism may involve oxidative stress produced by visceral adipose tissue, leading to disrupted collagen metabolism in the pelvic cavity and compromised support structures of the pelvic floor, thereby increasing the incidence and severity of incontinence [8]. However, body mass index (BMI) seems to be a low-sensitive indicator of abdominal obesity, as it fails to differentiate between adipose from non-adipose tissue, and does not account for variations in body fat distribution across different regions [9, 10]. Therefore, it is essential to combine body weight with waist circumference (WC) and height measurements to accurately assess abdominal obesity.

Specific anthropometric indices of abdominal obesity, such as body roundness index (BRI), waist-to-height ratio (WHtR), and conicity index (CI), have been developed to determine both the extent of abdominal adiposity and degree of abdominal obesity [11]. Previous evidence has established a connection between these anthropometric indices and various human diseases, including diabetes [12], cardiovascular diseases (CVD) [13], and gynecologic cancer [14]. However, limited research has examined their relationship with UI. A cross-sectional study revealed that measures such as body fat percentage, WC, waist-to-hip ratio, and relative fat mass are more effective indicators than BMI for assessing pelvic floor muscle distress [15]. Additionally, researchers discovered that a high BMI was associated with slight SUI, while WHtR consistently correlates with moderate MUI [16]. Importantly, due to variations in age groups, sample sizes, severity degrees of UI, and different anthropometric measurements used across studies, we cannot fully comprehend the relationship between specific abdominal obesity indices and UI and its severity.

In this study analyzing data from the National Health and Nutrition Examination Survey (NHANES) 2005–2018, we aim to examine the association between BRI, WHtR, and CI with the prevalence and severity of different types of UI, and to compare their predictive value with other commonly used anthropometric measures, such as BMI and WC. To our knowledge, this is the first study to explore the relationship between these novel anthropometric indices and UI in a nationwide prospective cohort.

## Methods

### Study population

NHANES ( http://www.cdc.gov/nchs/nhanes.htm) is an ongoing cross-sectional survey that aims to assess the health and nutritional status of the U.S. population, who are civilian and noninstitutionalized. This nationally representative survey utilizes a complex, multistage probability design to generate a sample of residents from all 50 states and Washington D.C. To obtain the required data, participants are randomly selected for household interviews, physical examinations, and laboratory tests every two years. The technical aspects of the sampling methodology and data collection have been detailed in a previous publication [17]. Conducted by the National Center for Health Statistics (NCHS), which is part of the Centers for Disease Control and Prevention (CDC), NHANES ensures the protection of human research subjects by requiring all participants to provide written informed consent.

**Fig. 1:**
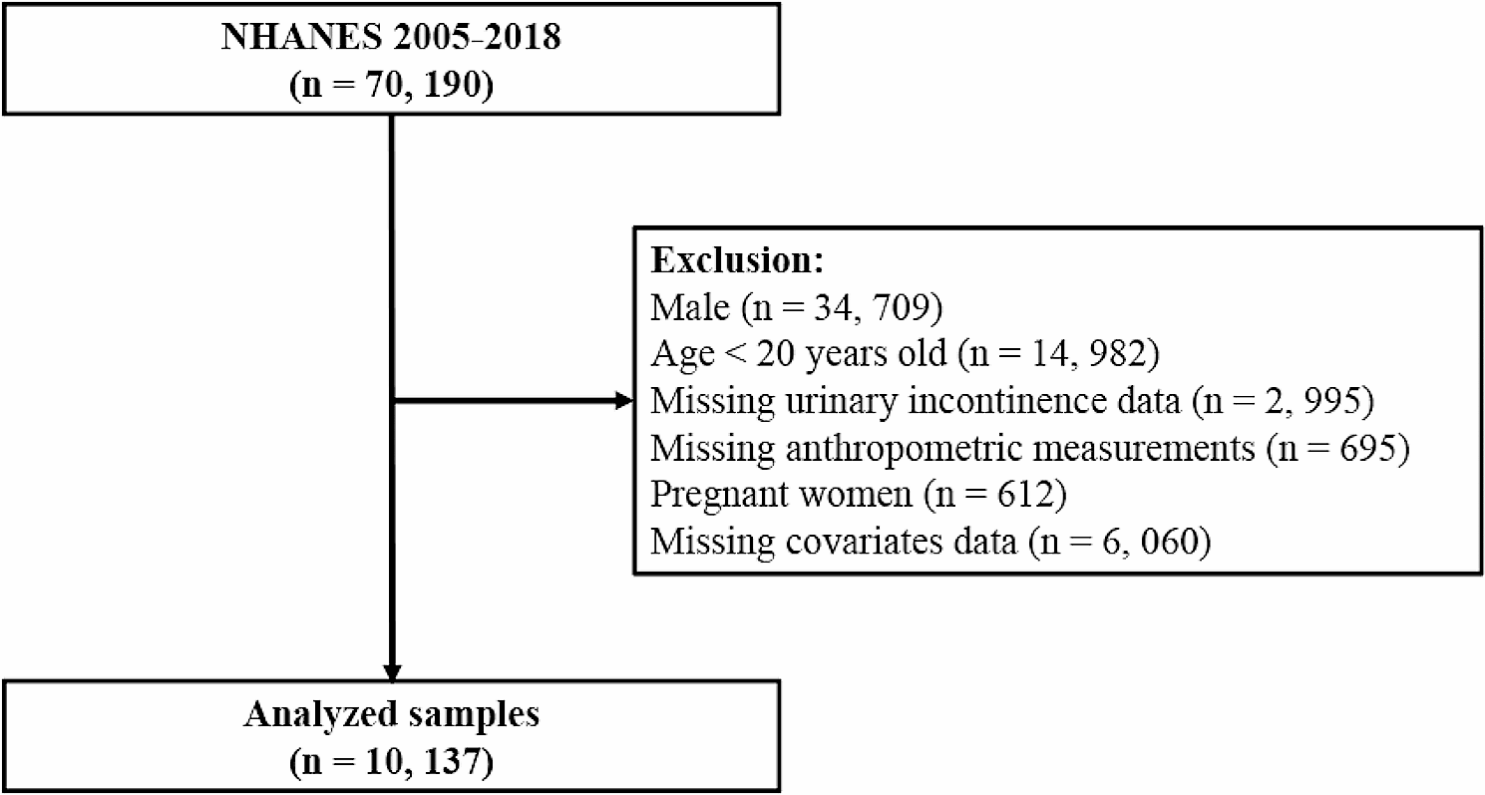
Flowchart of the study population from NHANES (2005–2018). NHANES National Health and Nutrition Examination Survey

In this study, we utilized publicly available data from NHANES waves spanning 2005–2006, 2007–2008, 2009–2010, 2011–2012, 2013–2014, 2015–2016, and 2017–2018, in accordance with relevant regulations and guidelines [17, 18]. Personally identifiable information was not included. A total of 35,888 participants were included in the study, who were aged ≥ 20 years old, completed questionnaires and examinations, and did not report pregnancy at the time of the survey (*n* = 612 excluded). Additionally, we excluded participants with missing UI and anthropometric measurements, reproductive health conditions, and chronic disease assessment data. After excluding individuals with missing information, the final analysis included a total of 10,317 female participants with complete interview and examination data (Fig. 1).

### Ethics approval

The authors take full responsibility for all aspects of the study and have taken steps to ensure that any questions related to the accuracy or integrity of the work are appropriately investigated and resolved. The research was conducted in accordance with the principles outlined in the Declaration of Helsinki (as revised in 2013). As all information from the NHANES program is publicly available, no approval from a medical ethics committee board was required. The study protocols for NHANES were approved by the NCHS ethnics review board (Protocol #2011–17, https://www.cdc.gov/nchs/nhanes/irba98.htm). Informed consent was obtained from all participants prior to their involvement in the study.

### Patient and public involvement

Patients or the public were not involved in the design, conduct, reporting or dissemination plans of our research.

### Urinary incontinence

The classification of UI was based on two questions from the “Kidney Condition-Urology” section of the survey. If participants answered positively to either question 1 (“During the past 12 months, have you leaked or lost control of even a small amount of urine with an activity like coughing, lifting, or exercise?“) or question 2 (“During the past 12 months, have you leaked or lost control of even a small amount of urine with an urge or pressure to urinate and couldn’t get to the toilet fast enough?“), they were classified as having UI. Those who only answered yes to question 1 were classified as having SUI, those who only answered yes to question 2 were classified as having UUI, and those who answered yes to both questions were classified as having MUI. The severity index for UI was assessed using the two-item Incontinence Severity Index [19] from the kidney condition questionnaire, which multiplied the frequency (four levels: less than once per month, a few times a month, a few times a week, and every day and/or night) and amount of urinary leakage (three levels: drops, splashes, or more) to obtain a score ranging from 1 to 12. The severity score for MUI was determined by taking the highest severity score between SUI and UUI from the same individual. A higher score indicated more severe symptoms. The UI severity score was categorized as ‘none’ (severity score = 0), ‘slight’ (severity score = 1–2), ‘moderate’ (severity score = 3–6), or ‘severe’ (severity score > 6) [19].

### Anthropometric measurements

The trunk fat ratio can, to some extent, reflect abdominal pressure and visceral obesity. The percentage of total and trunk body fat, including head, limbs, and trunk, was obtained from dual-energy x-ray absorptiometry-whole body scans performed during each NHANES survey cycle. The DXA scans were reviewed and analyzed by the Department of Radiology, University of California, San Francisco using standard radiologic techniques and NHANES-specific protocols. The Hologic Discovery software 12.1 was used to analyze the DXA exams and provide body composition data. By utilizing specific x-ray absorptivity of tissues with different densities, the DXA scan distinguishes fat tissue from other tissues and calculates the percentage of fat in the body. Fat percentages for only the trunk area were derived to measure the magnitude and distribution of body fat. Participants with complete data for total and trunk body fat were included for further analysis.

Basic anthropometric measurements were measured by well-trained examiners at a mobile examination center after participants removed bulky clothing and shoes. Body weight, height, and WC were measured with calibrated equipment according to standard procedures. WC was measured at a level midway between the inferior margin of the last rib and the uppermost lateral iliac crest in standing position. BMI was calculated as an individual’s weight in kilograms divided by the square of their height in meters (kg/m²). BMI, BRI, CI, WHtR, and A Body Shape Index (ABSI) were calculated using published formulas [20, 21] and shown as followed:

$$\rm{BRI = }364.2 - 365.5 \times \sqrt {1 - {{\left( {\frac{{\frac{{wc(m)}}{{2\Pi }}}}{{0.5 \times BH(m)}}} \right)}^2}}$$

$$ {\rm CI }= \frac{WC \left(m\right)}{0.109 \times \sqrt{\frac{BW\left(kg\right)}{BH\left(m\right)}}}$$

$${\rm{WHtR }} = {\rm{ WC }}(cm)/{\rm{ BH }}(cm)$$

$$ {\rm ABSI } = \text{W}\text{C} \left(m\right){ /BMI}^{\frac{2}{3}} (\text{k}\text{g}/{m}^{2})\times {BH}^{\frac{1}{2}} \left(m\right)$$

### Demographic characteristics

Demographic data on age, gender, race/ethnicity, family income, education level, marital status, smoking status, drinking status, and disease status were collected from household interviews using standardized questionnaires. Race was categorized as non-Hispanic white, non-Hispanic black, Mexican American, or other. Family income was categorized as ≤ $24,999, $25,000–54,999, $55,000–99,999, or ≥ $100,000. The family poverty income ratio (PIR) was categorized as < 1.3, 1.3–3.5, or ≥ 3.5. Education level was classified as less than high school, high school diploma, more than high school diploma. Marital status was categorized as married (including married and living with partner), never married, and separated (including widowed, divorced, and separated). Physical activity (PA) was calculated as the sum time of walking, moderate and vigorous activity undertaken in a week, and was categorized according to the level of metabolic equivalent of task (MET): low (0–499 MET-min activity/week), moderate (500–1,000 MET-min activity/week), and high (> 1,000 MET-min activity/week). Diabetes was defined as a self-reported doctor diagnosis of DM, glycated hemoglobin A1c (HbA1c) ≥ 6.5%, use of insulin or anti-diabetes drugs, fasting glucose ≥ 7.0 mmol/L, random glucose ≥ 11.1 mmol/L, or oral glucose tolerance test (OGTT) ≥ 11.1 mmol/L [22]. CVD was determined by a composite of a self-reported physician diagnosis with a standardized health condition or from the medical history questionnaire administered during individual interviews [23]. A positive response to any of five separate questions, regard to congestive heart failure, coronary heart disease, angina, heart attack, and stroke, indicated that the individual was considered positive for CVD. The reproductive health conditions include menstrual history (yes or no), pregnancy history (yes or no) was assessed with standardized questionnaires. Gynecological cancer was defined as any occurrence of cervical, ovarian, or endometrial cancer reported by participants in response to the Medical Status Questionnaire. This questionnaire included questions such as, “Have you ever been told by a doctor or other health professional that you had cancer or malignancy?” and “What kind of cancer was it?“.

### Statistical analysis

Sample weights, clusters, and stratification were incorporated into all analyses because of the complex sampling design of the NHANES, as required to analyze the NHANES data. According to the NHANES analytic guidelines, the appropriate survey weight is based on the variable of interest that was collected on the smallest number of respondents. Thus, the sample weight for seven cycles of NHANES was calculated by dividing the original 2-year sample weight by 7 and then assigning this weight to each participant. In addition, the variables SDMVPSU and SDMVSTRA were used to properly estimate the variance.

Continuous variables were presented as weighted means (standard errors), while categorical variables were presented as frequencies (weighted percentages). To evaluate differences between groups, various statistical tests were utilized. For categorical variables, weighted chi-square tests were performed. Weighted analysis of variance was used for normally distributed continuous variables, while the weighted Kruskal-Wallis H test was employed for skewed distributed continuous variables. The anthropometric measurements were converted into categorical variables by quartiles to explore the correlation and potential relationship with UI. Survey-weighted multiple logistic regression analysis was used to examine the association between various anthropometric measurements and the risk of UI.

To estimate potential differences in the confounding effects, we adjusted for several covariates, including year cycle, age categories, education, race, marital, PIR, alcohol drinking status, smoking status, PA. diabetes, CVD, gynecological cancer, pregnancy history and menstrual, in the full adjusted model. To analyze possible associations between anthropometric measurements and UI, we estimated adjusted odds ratios (ORs) and 95% confidence intervals (CIs). Moreover, stratified analyses were performed to investigate the interaction between BRI, WHtR, and CI and three types of UI. Receiver operating characteristic (ROC) curves were employed to assess the discriminative power of anthropometric measurements in identifying individuals with UI. DeLong’s test was used to compared the AUCs of two correlated ROC curves. All statistical analyses were conducted in R software (version 4.1.2; R Foundation for Statistical Computing, Vienna, Austria) using the package “survey” to account for the complex sampling design. Two-sided *P* < 0.05 was considered statistically significant.

## Results

### Population characteristics

Table 1 displays the baseline demographic characteristics of our study population, which included 10,137 female participants with a mean age of 46.4 years old. Out of this total population, a total of 2, 737 (27.0%) females were diagnosed with UUI, 4, 158 (41.0%) females were diagnosed with SUI, and 1,645 (16.2%) females were diagnosed with MUI. Our analysis revealed that females diagnosed with MUI had distinct baseline demographic characteristics compared to those without MUI. Specifically, females with MUI tended to be older, have lower education levels, higher prevalence of separation or divorce, less physical activity, and lower family income. Furthermore, our study found that participants with MUI had a higher ratio of pregnancy history, gynecological cancer history, diabetes, and CVD, especially in those at higher possible in postmenopausal, which is similar to females with SUI or UUI (Supplementary Tables 1 and Supplementary Table 2). Six often used anthropometric parameters involved in our study, including BMI, WC, BRI, WHtR, CI, and ABSI, were all higher in females with UI (including SUI, UUI, and MUI) (all *P* < 0.0001). For indicators of abdominal obesity, trunk fat ratio was also significantly higher in female with MUI than in females without MUI.

**Table 1:** Characteristics of non-weighted study participants according to MUI, NHANES 2005 to 2018 (*n* = 10, 137)

Characteristics	Total (*n* = 10, 137)	Non-MUI (*n* = 8, 492)	MUI (*n* = 1, 645)	*P* value
Age (years)				< 0.0001
20–40	3946(38.93)	3611(42.96)	335(20.31)	
41–60	3587(35.39)	2905(37.43)	682(44.97)	
≥61	2604(25.69)	1976(19.61)	628(34.72)	
Race				0.054
Non-Hispanic White	4642(45.79)	3840(71.10)	802(73.78)	
Non-Hispanic Black	2061(20.33)	1752(10.48)	309(9.77)	
Mexican American	1409(13.9)	1147(6.43)	262(6.68)	
Others	2025(19.98)	1753(11.99)	272(9.77)	
Education levels				< 0.0001
Less than high school	1819(17.94)	1425(10.42)	394(16.18)	
High school diploma	2109(20.8)	1727(19.91)	382(23.48)	
More than high school	6209(61.25)	5340(69.67)	869(60.34)	
Marriage status				< 0.0001
Never married	1946(19.2)	1741(18.58)	205(9.98)	
Separated	2577(25.42)	1997(19.84)	580(29.56)	
Married	5614(55.38)	4754(61.57)	860(60.46)	
Family income				< 0.0001
< $25,000	2322(22.91)	1854(15.46)	468(19.67)	
$25,000-$54,999	3917(38.64)	3260(33.88)	657(37.14)	
$55,000-$99,999	2344(23.12)	2006(28.13)	338(26.98)	
≥ $100,000	1554(15.33)	1372(22.53)	182(16.21)	
Family PIR				< 0.0001
< 1.3	3738(36.87)	3127(34.38)	611(37.45)	
1.3–3.5	2987(29.47)	2407(18.81)	580(23.28)	
≥ 3.5	3412(33.66)	2958(46.81)	454(39.27)	
Alcohol drinking status				0.151
Never	1841(18.16)	1547(19.63)	294(18.07)	
Moderate	5199(51.29)	4386(57.05)	813(55.87)	
Heavy	3097(30.55)	2559(23.32)	538(26.06)	
Smoking status				< 0.0001
Never	6450(63.63)	5531(62.68)	919(53.50)	
Current	1793(17.69)	1426(16.90)	367(22.44)	
Former	1894(18.68)	1535(20.42)	359(24.05)	
Physical activity				< 0.0001
Low	2540(25.06)	2061(23.20)	479(28.72)	
Moderate	1717(16.94)	1443(16.83)	274(17.21)	
High	5880(58.01)	4988(59.97)	892(54.07)	
Pregnant history (yes, %)	8284(81.72)	6782(77.00)	1502(90.18)	< 0.0001
Menopause (yes, %)	4813(47.48)	3741(42.77)	1072(64.21)	< 0.0001
Gynecological cancer (yes, %)	140(1.38)	94(1.35)	46(3.28)	< 0.0001
Diabetes (yes, %)	1465(14.45)	1091(9.39)	374(18.13)	< 0.0001
Cardiovascular disease (yes, %)	683(6.74)	468(4.42)	215(11.61)	< 0.0001
BMI, kg/m^2^	28.650(0.118)	28.276(0.123)	30.732(0.238)	< 0.0001
Waist circumference, cm	95.324(0.274)	94.342(0.288)	100.795(0.499)	< 0.0001
ABSI	0.080(0.000)	0.080(0.000)	0.081(0.000)	< 0.0001
BRI	5.357(0.040)	5.203(0.043)	6.218(0.080)	< 0.0001
CI	1.286(0.002)	1.280(0.002)	1.317(0.003)	< 0.0001
WHtR	0.587(0.002)	0.581(0.002)	0.624(0.003)	< 0.0001
Trunk fat ratio	0.450(0.002)	0.447(0.002)	0.467(0.003)	< 0.0001

Data were presented as the mean ± standard error (continuous) or number with percent (categorical). BMI, body mass index; PIR, poverty-income ratio; WHtR, waist-to-height ratio; CI, conicity index; ABSI, a body shape index; BRI, body round index

### Correlations between six anthropometric measures and abdominal obesity indices

Spearman rank correlation was conducted to explore the associations between six anthropometric measures and abdominal obesity indictor (Supplementary Table 3). BMI was strongly associated with BRI, WHtR, and waist (all *r* > 0.900), and mediately correlated with CI (*r* = 0.502), but did not correlate with ABSI. Although there is a high correlation between BMI and total fat and trunk fat, the correlation with trunk fat ratio is significantly reduced. This suggests that BMI may not be a satisfactory indicator for abdominal obesity. Moreover, CI had the highest correlation with trunk fat ratio, followed by WHtR and BRI (all *r* > 0.600). Therefore, these three indicators may be better indicators of abdominal obesity.

### Associations of BRI, WHtR, and CI with UI risk

Correlation analysis between BRI, WHtR, and CI and risk of UI using progressively adjusted multivariate regression were conducted with the first quartile interval of anthropometric measures as a control, respectively (Table 2). In the crude model, three anthropometric measures were positively correlated with the prevalence of MUI, UUI, and SUI, and three types of UI risk increased with increasing three anthropometric measures (*P* for trend < 0.0001). After full adjusting for cofounders of age, race, education, marital, poverty-income ratio, alcohol drinking status, smoking status, physical activity, diabetes, cardiovascular diseases, gynecological cancer, pregnancy history and menstrual, BRI was significantly correlated with increased risk of UUI (OR Q4 vs. Q1: 1.93, 95% CI 1.61–2.30, *P* < 0.0001, *P* for trend < 0.0001), SUI (OR Q4 vs. Q1: 2.29, 95% CI 1.94–2.70, *P* < 0.0001, *P* for trend < 0.0001), and MUI (OR Q4 vs. Q1: 2.26, 95% CI 1.82–2.82, *P* < 0.0001, *P* for trend < 0.0001), which similar correlation were found in WHtR, CI, and BMI (all *P* < 0.001).

**Table 2:** Multivariable weighted odds ratios (ORs) with 95% confidence intervals (CIs) for the association between anthropometric indices and UI in US population, NHANES 2005–2018

Characteristics	Q1	Q2		Q3		Q4		*P* for trend
		OR (95% CI)	*P* value	OR (95% CI)	*P* value	OR (95% CI)	*P* value	
**CI**								
UUI								
Crude model	Reference	1.58(1.31,1.90)	< 0.0001	2.12(1.80,2.50)	< 0.0001	2.75(2.33,3.25)	< 0.0001	< 0.0001
Full adjusted*	Reference	1.25(1.03,1.52)	0.020	1.38(1.16,1.64)	< 0.001	1.48(1.24,1.78)	< 0.0001	< 0.0001
SUI								
Crude model	Reference	1.46(1.27,1.67)	< 0.0001	2.16(1.86,2.51)	< 0.0001	2.31(1.97,2.71)	< 0.0001	< 0.0001
Full adjusted*	Reference	1.19(1.03,1.38)	0.017	1.63(1.38,1.92)	< 0.0001	1.61(1.35,1.93)	< 0.0001	< 0.0001
MUI								
Crude model	Reference	1.60(1.29,1.97)	< 0.0001	2.34(1.89,2.90)	< 0.0001	2.87(2.33,3.52)	< 0.0001	< 0.0001
Full adjusted*	Reference	1.24(0.99,1.55)	0.064	1.51(1.20,1.90)	< 0.001	1.54(1.23,1.94)	< 0.001	< 0.0001
**BRI**								
UUI								
Crude model	Reference	1.46(1.23,1.74)	< 0.0001	2.05(1.73,2.42)	< 0.0001	2.87(2.44,3.36)	< 0.0001	< 0.0001
Full adjusted*	Reference	1.12(0.92,1.36)	0.250	1.41(1.18,1.69)	< 0.001	1.93(1.61,2.30)	< 0.0001	< 0.0001
SUI								
Crude model	Reference	1.75(1.50,2.05)	< 0.0001	2.01(1.74,2.32)	< 0.0001	2.68(2.30,3.11)	< 0.0001	< 0.0001
Full adjusted*	Reference	1.50(1.27,1.77)	< 0.0001	1.63(1.39,1.92)	< 0.0001	2.29(1.94,2.70)	< 0.0001	< 0.0001
MUI								
Crude model	Reference	1.65(1.36,2.01)	< 0.0001	2.15(1.75,2.65)	< 0.0001	3.30(2.71,4.02)	< 0.0001	< 0.0001
Full adjusted*	Reference	1.29(1.04,1.58)	0.019	1.50(1.20,1.88)	< 0.001	2.26(1.82,2.82)	< 0.0001	< 0.0001
**WHtR**								
UUI								
Crude model	Reference	1.46(1.23,1.74)	< 0.0001	2.04(1.72,2.41)	< 0.0001	2.86(2.44,3.35)	< 0.0001	< 0.0001
Full adjusted*	Reference	1.12(0.92,1.36)	0.248	1.41(1.18,1.68)	< 0.001	1.92(1.61,2.29)	< 0.0001	< 0.0001
SUI								
Crude model	Reference	1.74(1.50,2.03)	< 0.0001	2.01(1.74,2.33)	< 0.0001	2.66(2.29,3.10)	< 0.0001	< 0.0001
Full adjusted*	Reference	1.50(1.27,1.76)	< 0.0001	1.63(1.38,1.93)	< 0.0001	2.27(1.93,2.68)	< 0.0001	< 0.0001
MUI								
Crude model	Reference	1.65(1.36,2.00)	< 0.0001	2.16(1.75,2.66)	< 0.0001	3.28(2.70,3.99)	< 0.0001	< 0.0001
Full adjusted*	Reference	1.28(1.04,1.58)	0.019	1.51(1.20,1.89)	< 0.001	2.24(1.80,2.80)	< 0.0001	< 0.0001
**BMI**								
UUI								
Crude model	Reference	1.32(1.12,1.56)	0.001	1.90(1.62,2.24)	< 0.0001	2.23(1.91,2.61)	< 0.0001	< 0.0001
Full adjusted*	Reference	1.13(0.95,1.35)	0.163	1.50(1.26,1.79)	< 0.0001	1.85(1.55,2.20)	< 0.0001	< 0.0001
SUI								
Crude model	Reference	1.44(1.24,1.67)	< 0.0001	1.80(1.54,2.10)	< 0.0001	2.26(1.93,2.66)	< 0.0001	< 0.0001
Full adjusted*	Reference	1.33(1.14,1.55)	< 0.001	1.56(1.32,1.84)	< 0.0001	2.19(1.83,2.62)	< 0.0001	< 0.0001
MUI								
Crude model	Reference	1.36(1.12,1.65)	0.003	1.85(1.51,2.26)	< 0.0001	2.42(2.02,2.90)	< 0.0001	< 0.0001
Full adjusted*	Reference	1.17(0.96,1.43)	0.115	1.46(1.18,1.80)	< 0.001	2.02(1.65,2.46)	< 0.0001	< 0.0001

*Full adjusted for year cycle, age categories, education, race, marital, poverty-income ratio, alcohol drinking status, smoking status, physical activity, diabetes, cardiovascular diseases, gynecological cancer, pregnancy history and menstrual

SUI, stress urinary incontinence; UUI, urge urinary incontinence; MUI, mixed urinary incontinence; WHtR, waist-to-height ratio; CI, conicity index; BRI, body round index; BMI, body mass index

### Association of BRI, WHtR, and CI with the UI severity index

As shown in the Table 3, the incontinence severity index of UUI would significantly increase 1.05 (95% CI 0.56–1.54, *P* < 0.001) with a one-unit increment of CI in the fully adjusted model. The tendency was similar in SUI (β: 2.07, 95% CI 1.46–2.68, *P* < 0.001), and MUI (β: 1.10, 95% CI 0.58–1.61, *P* < 0.0001). Similarly, a one-unit increment of BRI was significantly associated with an increased severity index of UUI (β: 0.06, 95% CI 0.04–0.09, *P* < 0.0001), SUI (β: 0.10, 95% CI 0.07–0.13, *P* < 0.0001) and MUI (β: 0.07, 95% CI 0.04–0.10, *P* < 0.0001). As expected, this trend was also observed in each subtype of UI for WHtR [UUI (β: 1.50, 95% CI 1.00-201, *P* < 0.0001), SUI (β: 2.41, 95% CI 1.74–3.07, *P* < 0.0001) and MUI (β: 1.58, 95% CI 0.99–2.18, *P* < 0.0001), respectively]. Moreover, elevated levels of CI, WHtR, BRI, and BMI were found to have a significant correlation with moderate or more severe UI. Notably, higher WHtR levels exhibited the greatest risk for developing moderate or more severe UI (UUI: OR 21.97, 95% CI 8.55–56.42, *P* < 0.0001; SUI: OR 29.06, 95% CI 10.25–82.40, *P* < 0.0001; MUI: OR 43.19, 95% CI 14.70-126.88, *P* < 0.0001), and BMI exhibited the lowest risk for developing moderate or more severe UI (UUI: OR 1.04, 95% CI 1.03–1.05, *P* < 0.0001; SUI: OR 1.04, 95% CI 1.03–1.06, *P* < 0.0001; MUI: OR 1.05, 95% CI 1.03–1.06, *P* < 0.0001).

**Table 3:** Associations between anthropometric indices and severity of UI in US population, NHANES 2005–2018

Characteristics	UI severity score*		Moderate or more severe UI#	
	β (95% CI)	*P* value	OR (95% CI)	*P* value
**CI**				
UUI	1.05(0.56, 1.54)	< 0.0001	11.02(3.83,31.67)	< 0.0001
SUI	2.07(1.46, 2.68)	< 0.0001	26.66(9.57,74.26)	< 0.0001
MUI	1.10(0.58, 1.61)	< 0.0001	17.11(5.60,52.25)	< 0.0001
**BRI**				
UUI	0.06(0.04, 0.09)	< 0.0001	1.13(1.09,1.17)	< 0.0001
SUI	0.10(0.07, 0.13)	< 0.0001	1.14(1.09,1.19)	< 0.0001
MUI	0.07(0.04, 0.10)	< 0.0001	1.16(1.11,1.21)	< 0.0001
**WHtR**				
UUI	1.50(1.00, 2.01)	< 0.0001	21.97(8.55,56.42)	< 0.0001
SUI	2.41(1.74, 3.07)	< 0.0001	29.06(10.25,82.40)	< 0.0001
MUI	1.58(0.99, 2.18)	< 0.0001	43.19(14.70,126.88)	< 0.0001
**BMI**				
UUI	0.02(0.01, 0.03)	< 0.0001	1.04(1.03,1.05)	< 0.0001
SUI	0.03(0.02, 0.04)	< 0.0001	1.04(1.03,1.06)	< 0.0001
MUI	0.02(0.01, 0.03)	< 0.0001	1.05(1.03,1.06)	< 0.0001

Full adjusted for year cycle, age categories, education, race, marital, poverty-income ratio, alcohol drinking status, smoking status, physical activity, diabetes, cardiovascular diseases, gynecological cancer, pregnancy history and menstrual

* UI severity score was measured as continuous variable

* Moderate or more severe UI was defined as the UI severity score ≥ 3 and compared with participants with UI severity score < 3

SUI, stress urinary incontinence; UUI, urge urinary incontinence; MUI, mixed urinary incontinence; WHtR, waist-to-height ratio; CI, conicity index; BRI, body round index; BMI, body mass index

### Stratification analysis based on menstruation

By using a stratified analysis in conjunction with full adjusted model, the stability of the correlation between CI, WHtR, BRI, and three types of UI risks were further confirmed in different populations (Table 4). As a result of stratification by menstruation, the highest quartile interval of CI was significantly correlated with UI risk in premenopausal females compared to the lowest quartile interval of CI (UUI: OR 1.56, 95% CI 1.18–2.07, *P* = 0.002; SUI: OR 1.79, 95% CI 1.41–2.27, *P* < 0.0001; MUI: OR 1.78, 95% CI 1.24–2.54, *P* = 0.002, respectively). However, CI was significantly correlated with UUI and SUI risk in postmenopausal females, but not for MUI. Moreover, there was an interaction between BRI, WHtR, BMI and menstrual for MUI risk (all *P* for interaction < 0.05). The risk of MUI in females without menstrual increased rapidly with the increase of these parameters, especial in the upper quantile (BRI: OR Q4 vs. Q1: 3.02, 95% CI 2.14–4.26, *P* < 0.0001; WHtR: OR Q4 vs. Q1: 3.00, 95% CI 2.13–4.23, *P* < 0.0001; respectively), but BRI and WHtR were statistically significant only when it was at high values in postmenopausal females.

**Table 4:** Stratification analysis for the association between the anthropometric indices and UI risk in participants with or without menstruation

Characters	Q1	Q2		Q3		Q4		*P* for interaction
		OR (95% CI)	*P* value	OR (95% CI)	*P* value	OR (95% CI)	*P* value	
**CI**								
Menstruation								
No								
UUI	Reference	1.28(1.00,1.64)	0.052	1.40(1.05,1.87)	0.023	1.56(1.18,2.07)	0.002	0.823
SUI	Reference	1.12(0.93,1.34)	0.230	1.73(1.39,2.14)	< 0.0001	1.79(1.41,2.27)	< 0.0001	0.210
MUI	Reference	1.33(0.95,1.84)	0.093	1.73(1.19,2.52)	0.004	1.78(1.24,2.54)	0.002	0.319
Yes								
UUI	Reference	1.18(0.86,1.61)	0.306	1.29(0.98,1.70)	0.071	1.38(1.03,1.84)	0.031	0.823
SUI	Reference	1.30(0.98,1.71)	0.067	1.51(1.15,2.00)	0.004	1.49(1.12,1.98)	0.007	0.210
MUI	Reference	1.08(0.74,1.56)	0.690	1.25(0.88,1.78)	0.209	1.29(0.91,1.83)	0.151	0.319
**BRI**								
Menstruation								
No								
UUI	Reference	1.31(0.98,1.74)	0.068	1.43(1.07,1.91)	0.016	2.22(1.70,2.91)	< 0.0001	0.163
SUI	Reference	1.43(1.16,1.76)	< 0.001	1.78(1.43,2.21)	< 0.0001	2.45(1.96,3.05)	< 0.0001	0.443
MUI	Reference	1.42(1.01,2.00)	0.042	1.68(1.19,2.38)	0.004	3.02(2.14,4.26)	< 0.0001	0.050
Yes								
UUI	Reference	0.96(0.74,1.23)	0.720	1.32(1.04,1.68)	0.023	1.67(1.29,2.16)	< 0.001	0.163
SUI	Reference	1.54(1.17,2.03)	0.002	1.49(1.14,1.95)	0.004	2.13(1.61,2.81)	< 0.0001	0.443
MUI	Reference	1.12(0.82,1.52)	0.462	1.30(0.97,1.76)	0.083	1.78(1.31,2.43)	< 0.001	0.049
**WHtR**								
Menstruation								
No								
UUI	Reference	1.30(0.98,1.74)	0.070	1.44(1.08,1.92)	0.014	2.21(1.69,2.89)	< 0.0001	0.180
SUI	Reference	1.43(1.16,1.76)	0.001	1.79(1.44,2.22)	< 0.0001	2.44(1.95,3.04)	< 0.0001	0.390
MUI	Reference	1.42(1.01,2.00)	0.045	1.69(1.20,2.40)	0.003	3.00(2.13,4.23)	< 0.0001	0.049
Yes								
UUI	Reference	0.96(0.74,1.23)	0.729	1.31(1.03,1.66)	0.029	1.66(1.29,2.15)	< 0.001	0.180
SUI	Reference	1.54(1.18,2.02)	0.002	1.48(1.13,1.95)	0.005	2.10(1.60,2.78)	< 0.0001	0.390
MUI	Reference	1.12(0.83,1.52)	0.461	1.30(0.96,1.76)	0.084	1.76(1.29,2.40)	< 0.001	0.047
**BMI**								
Menstruation								
No								
UUI	Reference	1.37(1.01,1.86)	0.043	1.49(1.12,2.00)	0.008	2.19(1.67,2.86)	< 0.0001	0.072
SUI	Reference	1.41(1.13,1.76)	0.003	1.68(1.33,2.11)	< 0.0001	2.33(1.83,2.97)	< 0.0001	0.711
MUI	Reference	1.36(0.94,1.96)	0.098	1.49(1.05,2.12)	0.024	2.62(1.88,3.65)	< 0.0001	0.047
Yes								
UUI	Reference	0.98(0.78,1.23)	0.851	1.49(1.20,1.84)	< 0.001	1.61(1.27,2.04)	< 0.001	0.072
SUI	Reference	1.25(0.97,1.61)	0.083	1.43(1.12,1.84)	0.005	2.06(1.59,2.66)	< 0.0001	0.711
MUI	Reference	1.06(0.82,1.37)	0.671	1.40(1.09,1.81)	0.010	1.66(1.28,2.14)	< 0.001	0.047

Full adjusted for year cycle, age categories, education, race, marital, poverty-income ratio, alcohol drinking status, smoking status, physical activity, diabetes, cardiovascular diseases, gynecological cancer, and pregnancy history

SUI, stress urinary incontinence; UUI, urge urinary incontinence; MUI, mixed urinary incontinence; WHtR, waist-to-height ratio; CI, conicity index; BRI, body round index; BMI, body mass index

### Discrimination ability of different anthropometric measures

Given that BMI and WC are the most widely used anthropometric indices, while commonly used indices reflecting abdominal obesity are CI, BRI, and WHtR, as well as trunk fat ratio. Therefore, ROC curves and area under the curve (AUC) were performed to evaluate the abilities of different anthropometric measures in discriminating females with UI (Supplementary Fig. 1). The results of Delong’s test demonstrated a higher diagnostic efficacy of BRI and WHtR in discriminating UI with an AUC of 0.600 for SUI, 0.617 for UUI, and 0.622 for MUI, which compared with BMI (all *P* < 0.05).

## Discussion

In this nationwide prospective cohort study with 10, 137 female participants from seven consecutive NHANES 2-year cycles spanning from 2005 to 2018, we found that BRI, CI, and WHtR were the three most strongly correlated with trunk fat ratio, a representative abdominal obesity index. After adjusting for all relevant confounding variables, we observed a positive association between BRI, CI, WHtR, and the prevalence of all three types of UI, which particularly prominent in female individuals without postmenopausal. A higher index of BRI, CI, and WHtR suggest a more severe degree of incontinence. Moreover, BRI, CI, and WHtR had a better ability to predict three types of UI risk than BMI, WC, ABSI, and trunk fat ratio.

Accumulating evidence has supported the leading role of obesity in the pathogenesis of UI [6]. From an anatomical perspective, abdominal obesity leads to an increase in intra-abdominal pressure, which weakens the pelvic muscles and nerve innervation, resulting in reduced pelvic floor muscle strength and UI symptoms [24]. Pathophysiologically, the accumulation of visceral fat tissue results in dysregulation of various inflammatory cytokines, further activating oxidative stress and leading to metabolic disorders and collagen metabolism irregularities in human pelvic fibroblasts, ultimately increasing the incidence and severity of UI [25]. Furthermore, UI symptoms often reduce physical activity levels, causing fat accumulation and loss of skeletal muscle mass, further increasing the risk of hospitalization for UI patients [26]. Abdominal obesity, particularly the accumulation of fat in deep subcutaneous tissue, has been identified as a key driving factor for the progression of UI, insulin resistance, and cardiovascular events. Therefore, BMI, using total body weight, may not accurately reflect an individual’s obesity status, particularly for UI patients. In contrast, BRI, CI, and WHtR are calculated by combining waist circumference with body weight and height measurements, which better reflect central obesity and can assess high fat mass and low muscle mass. In present study, we found that these three anthropometric measurements have the strongest correlation with trunk fat ratio and significant positive correlations with the three types of UI.

Previous studies have consistently demonstrated that females with abdominal obesity was associated with a higher prevalence and severity of UI [27, 28]. Meanwhile, female patients with SUI had a higher obesity tendency [29]. The results of a large prospective cohort study also support our findings that SUI odds ratios increased significantly in a dose-dependent manner with the next quartile of waist circumference [27]. In a 4-year trial with 2, 763 postmenopausal women, the results indicated that an increasing waist-to-hip ratio was an independent risk factor for SUI (OR 1.18 per 0.1-unit increase), but not for UUI and MUI [30]. In a 2-year follow-up study of elderly women in the Nurses’ Health Study, the inclusion of both BMI and waist circumference in the model revealed that BMI was associated with UUI and MUI, but not with SUI. However, WC, a surrogate index for abdominal obesity, was only associated with SUI [31]. Our study complements previously reported evaluations of obesity indicators by showing that BRI, CI, and WHtR was superior to BMI and WC for evaluating the risk of three types of UI, including UUI, SUI, and MUI. After adjusting for pregnant history, gynecological cancer, and menopausal, the prevalence of UI increased significantly in a dose dependent relationship in the higher quartiles of BRI, CI, and WHtR, which was also associated with the severity of UI.

Our study found that index of abdominal obesity, such as BRI, CI, and WHtR, were associated with the prevalence and severity of UI risks. Stratified analyses indicated that the positive association between the index of abdominal obesity and UI risk was more pronounced among those females without menstruation at baseline. This suggests that interventions focusing on optimal body shape control may be more effective at the time of premenopausal for UI prevention. Menopause and aging are closely associated with the onset or aggravation of lower urinary tract dysfunction [32]. The effect of menopause can be partly explained by the impact of estrogen withdrawal on collagen remodeling, which negatively affects urethral mobility and closure mechanisms, as well as an increase in collagen content in the detrusor smooth muscle, which may exacerbate symptoms of overactive bladder and lead to the development of UI [33, 34]. Numerous studies have suggested that obesity is linked to a later onset of menopause [35, 36]. One plausible explanation is that androgens are converted peripherally to estrogens in adipose tissue in a high level, which leads to a delay in the onset of menopause and ultimately manifests as estrogen deficiency [37]. Recent meta-analyses provide evidence supporting the use of local estrogen therapy in postmenopausal women to improve UI symptoms, and this treatment approach is both safe and effective [38]. Therefore, obesity and menopause may have a synergistic effect on UI through interact pathways, which also been indicated in our findings that the significant interaction correlation was found between CI, WHtR, and menopause for MUI risks.

Many studies have demonstrated that indices of abdominal obesity (BRI, CI, and WHtR) are reliable predictors of metabolic syndrome, which is related to cardiometabolic risk and metabolic-associated fatty liver disease risks [39–41]. Additionally, previous study found that SUI and UUI are more prevalent in pre- and postmenopausal women with metabolic syndrome [42, 43]. Given these findings, prevention of UI may be achieved through lifestyle changes and management of the body shape to reduce the development of metabolic syndrome. Regular physical activity and weight management can help mitigate the incidence and severity of UI, while monitoring BRI, CI, and WHtR levels at periodic intervals can aid in evaluating the efficacy of lifestyle interventions and guiding ongoing treatment strategies.

Several limitations of the present study should be noted. First, although survey-weighted multiple logistic regression analysis adjusted for the covariates, a lack of adjustment for residual and unmeasured confounds could also produce a bias. Second, the cross-sectional nature of our study prevented us from establishing a temporal relationship among BRI, CI, WHtR, and UI; thus, the associations cannot be interpreted as causal relations. There is a possibility that participants may have experienced UI symptoms before developing abdominal obesity. These symptoms may have led to a decrease in physical activity, which in turn may have facilitated the development of abdominal obesity. If high values of BRI, CI, and WHtR are systematically documented, this may increase the likelihood of an existing urinary incontinence (UI) diagnosis. As a result, this could potentially inflate the observed associations between these factors. Third, while the sample size was large, our participants were limited to U.S. residents who were willing to participate in the study, and the generalizability of our results to the rest of the U.S. and other countries is unknown.

## Conclusions

In summary, an increased BRI, CI, and WHtR are significantly associated with higher prevalence and severity of UI in females. It suggested that it may potentially be used as a simple anthropometric index to predict UI.

### Electronic supplementary material

Below is the link to the electronic supplementary material.

Supplementary Material 1

Supplementary Material 2

Supplementary Material 3

Supplementary Material 4

## Data Availability

The National Health and Nutrition Examination Survey (NHANES) data are publicly available at https://www.cdc.gov/nchs/nhanes/index.htm.

## References

[1] Rogers RG, Pauls RN, Thakar R, Morin M, Kuhn A, Petri E (2018). An international Urogynecological Association (IUGA)/international continence society (ICS) joint report on the terminology for the assessment of sexual health of women with pelvic floor dysfunction. Int Urogynecol J.

[2] Przydacz M, Chlosta M, Chlosta P. Population-Level Prevalence, Bother, and treatment behavior for urinary incontinence in an eastern European Country: findings from the LUTS POLAND Study. J Clin Med 2021;10(11). 10.3390/jcm10112314 PMC8199423 34073165

[3] Abufaraj M, Xu T, Cao C, Siyam A, Isleem U, Massad A (2021). Prevalence and trends in urinary incontinence among women in the United States, 2005–2018. Am J Obstet Gynecol.

[4] Lee UJ, Feinstein L, Ward JB, Kirkali Z, Martinez-Miller EE, Matlaga BR (2021). Prevalence of urinary incontinence among a nationally Representative Sample of women, 2005–2016: findings from the urologic diseases in America Project. J Urol.

[5] Legendre G, Fritel X, Panjo H, Zins M, Ringa V (2020). Incidence and remission of stress, urge, and mixed urinary incontinence in midlife and older women: a longitudinal cohort study. Neurourol Urodyn.

[6] Fuselier A, Hanberry J, Margaret LJ, Gomelsky A (2018). Obesity and stress urinary incontinence: impact on pathophysiology and treatment. Curr Urol Rep.

[7] Subak LL, King WC, Belle SH, Chen JY, Courcoulas AP, Ebel FE (2015). Urinary incontinence before and after bariatric surgery. Jama Intern Med.

[8] Doumouchtsis SK, Loganathan J, Pergialiotis V (2022). The role of obesity on urinary incontinence and anal incontinence in women: a review. BJOG.

[9] Okorodudu DO, Jumean MF, Montori VM, Romero-Corral A, Somers VK, Erwin PJ (2010). Diagnostic performance of body mass index to identify obesity as defined by body adiposity: a systematic review and meta-analysis. Int J Obes (Lond).

[10] Rodrigues J, Santin F, Barbosa BF, Carrero JJ, Lindholm B, Cuppari L (2016). Sensitivity and specificity of body Mass Index as a marker of obesity in Elderly patients on Hemodialysis. J Ren Nutr.

[11] Piqueras P, Ballester A, Dura-Gil JV, Martinez-Hervas S, Redon J, Real JT (2021). Anthropometric indicators as a Tool for diagnosis of obesity and other Health risk factors: a Literature Review. Front Psychol.

[12] Zhao Q, Zhang K, Li Y, Zhen Q, Shi J, Yu Y (2018). Capacity of a body shape index and body roundness index to identify diabetes mellitus in Han Chinese people in Northeast China: a cross-sectional study. Diabet Med.

[13] Li Y, He Y, Yang L, Liu Q, Li C, Wang Y (2022). Body roundness Index and Waist-Hip ratio result in Better Cardiovascular Disease Risk Stratification: results from a large Chinese cross-sectional study. Front Nutr.

[14] Wilczynski M, Domanska-Senderowska D, Kassassir-Cwiklak SA, Janas L, Malinowski A, Wilczynski JR (2021). A body shape index (ABSI) and endometrial pathology. Women Health.

[15] Scarabelot KS, Da SPF, Pelegrini A, Tuon T, Virtuoso JF (2020). Anthropometric indicators as predictors of pelvic floor muscle distress in young women. Neurourol Urodyn.

[16] Li Y, Zhang Z (2017). Association between waist-to-height ratio and postpartum urinary incontinence. Int Urogynecol J.

[17] Johnson CL, Paulose-Ram R, Ogden CL, Carroll MD, Kruszon-Moran D, Dohrmann SM et al. National health and nutrition examination survey: analytic guidelines, 1999–2010. Vital Health Stat 2 2013(161):1–24. 25090154

[18] Health (1992). Population data for developing countries available through USAID project. J Trop Med Hyg.

[19] Sandvik H, Seim A, Vanvik A, Hunskaar S (2000). A severity index for epidemiological surveys of female urinary incontinence: comparison with 48-hour pad-weighing tests. Neurourol Urodyn.

[20] Su WY, Chen IH, Gau YC, Wu PY, Huang JC, Tsai YC et al. Metabolic syndrome and obesity-related indices are Associated with Rapid Renal function decline in a large Taiwanese Population Follow-Up study. Biomedicines 2022;10(7). 10.3390/biomedicines10071744 PMC9312807 35885048

[21] Wu LD, Kong CH, Shi Y, Zhang JX, Chen SL (2022). Associations between novel anthropometric measures and the prevalence of hypertension among 45,853 adults: a cross-sectional study. Front Cardiovasc Med.

[22] Wan Z, Guo J, Pan A, Chen C, Liu L, Liu G (2021). Association of serum 25-Hydroxyvitamin D concentrations with all-cause and cause-specific mortality among individuals with diabetes. Diabetes Care.

[23] Xu C, Liang J, Xu S, Liu Q, Xu J, Gu A (2020). Increased serum levels of aldehydes are associated with cardiovascular disease and cardiovascular risk factors in adults. J Hazard Mater.

[24] Padmanabhan P, Dmochowski R (2014). Urinary incontinence in women: a comprehensive review of the pathophysiology, diagnosis and treatment. Minerva Ginecol.

[25] Post WM, Widomska J, Grens H, Coenen M, Martens F, Janssen D et al. Molecular processes in stress urinary incontinence: a systematic review of Human and Animal studies. Int J Mol Sci 2022;23(6). 10.3390/ijms23063401 PMC8949972 35328824

[26] Kuutti MA, Hyvarinen M, Kauppinen M, Sipila S, Aukee P, Laakkonen EK (2023). Early adulthood and current physical activity and their association with symptoms of pelvic floor disorders in middle-aged women: an observational study with retrospective physical activity assessment. BJOG.

[27] Han MO, Lee NY, Park HS (2006). Abdominal obesity is associated with stress urinary incontinence in Korean women. Int Urogynecol J Pelvic Floor Dysfunct.

[28] Lai HH, Helmuth ME, Smith AR, Wiseman JB, Gillespie BW, Kirkali Z (2019). Relationship between central obesity, General Obesity, overactive bladder syndrome and urinary incontinence among male and female patients seeking care for their lower urinary tract symptoms. Urology.

[29] Stroher R, Sartori M, Takano CC, de Araujo MP, Girao M (2020). Metabolic syndrome in women with and without stress urinary incontinence. Int Urogynecol J.

[30] Brown JS, Grady D, Ouslander JG, Herzog AR, Varner RE, Posner SF (1999). Prevalence of urinary incontinence and associated risk factors in postmenopausal women. Heart & Estrogen/Progestin Replacement Study (HERS) Research Group. Obstet Gynecol.

[31] Townsend MK, Curhan GC, Resnick NM, Grodstein F (2008). BMI, waist circumference, and incident urinary incontinence in older women. Obes (Silver Spring).

[32] Monteleone P, Mascagni G, Giannini A, Genazzani AR, Simoncini T (2018). Symptoms of menopause - global prevalence, physiology and implications. Nat Rev Endocrinol.

[33] Radziszewski P, Borkowski A, Torz C, Bossowska A, Gonkowski S, Majewski M (2005). Distribution of collagen type VII in connective tissues of postmenopausal stress-incontinent women. Gynecol Endocrinol.

[34] Russo E, Caretto M, Giannini A, Bitzer J, Cano A, Ceausu I (2021). Management of urinary incontinence in postmenopausal women: an EMAS clinical guide. Maturitas.

[35] Appiah D, Nwabuo CC, Ebong IA, Wellons MF, Winters SJ (2021). Trends in Age at Natural Menopause and Reproductive Life Span among US women, 1959–2018. JAMA.

[36] Park CY, Lim JY, Park HY (2018). Age at natural menopause in koreans: secular trends and influences thereon. Menopause.

[37] Opoku AA, Abushama M, Konje JC (2023). Obesity and menopause. Best Pract Res Clin Obstet Gynaecol.

[38] Gartlehner G, Patel SV, Feltner C, Weber RP, Long R, Mullican K (2017). Hormone therapy for the primary Prevention of Chronic conditions in Postmenopausal women: evidence report and systematic review for the US Preventive Services Task Force. JAMA.

[39] Bialkowska A, Gornicka M, Zielinska-Pukos MA, Hamulka J. Associations between dietary patterns, anthropometric and cardiometabolic indices and the Number of MetS Components in Polish adults with metabolic disorders. Nutrients 2023;15(10). 10.3390/nu15102237 PMC10224261 37242120

[40] Wang H, Zhang Y, Liu Y, Li H, Xu R, Fu H (2023). Comparison between traditional and new obesity measurement index for screening metabolic associated fatty liver disease. Front Endocrinol (Lausanne).

[41] Sisay BG, Jima BR, Habtamu M, Gebru NW, Hassen HY (2023). Predictive ability of anthropometric indices in identifying metabolic syndrome among US adolescents 10 to 19 years old: analysis from the National Health and Nutrition Examination Survey 2011 to 2018 data set. Nutrition.

[42] Otunctemur A, Dursun M, Ozbek E, Sahin S, Besiroglu H, Koklu I (2014). Impact of metabolic syndrome on stress urinary incontinence in pre- and postmenopausal women. Int Urol Nephrol.

[43] Hsu LN, Hu JC, Chen PY, Lee WC, Chuang YC. Metabolic syndrome and overactive bladder syndrome may share common pathophysiologies. Biomedicines 2022;10(8). 10.3390/biomedicines10081957 PMC9405560 36009505

