# Supplementary figures and images for: Abdominal obesity as assessed by anthropometric measures associates with urinary incontinence in females: findings from the National Health and Nutrition Examination Survey 2005–2018
Source: BMC Womens Health. 2024 Apr 2;24:212. doi: 10.1186/s12905-024-03059-2 (PMC10986057; doi:10.1186/s12905-024-03059-2)

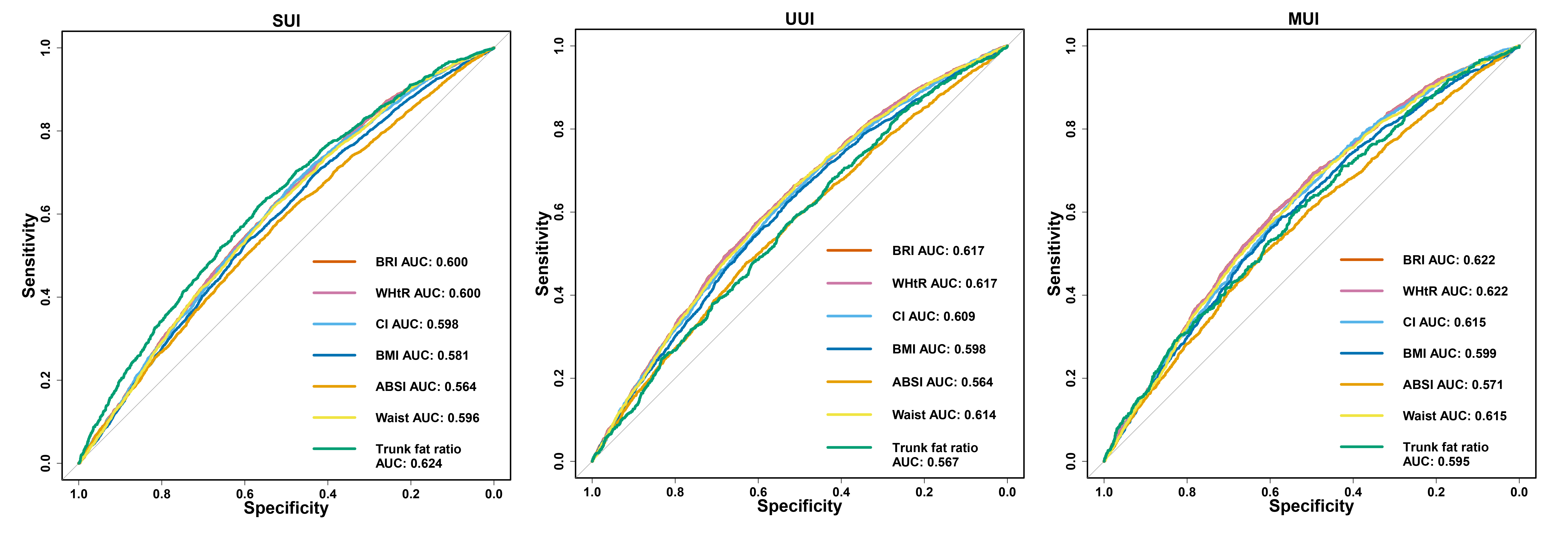

Supplement: Supplementary file 1 — Supplementary Material 1 [file 12905_2024_3059_MOESM1_ESM.tif]
