# Supplementary material for: Abdominal obesity as assessed by anthropometric measures associates with urinary incontinence in females: findings from the National Health and Nutrition Examination Survey 2005–2018
Source: BMC Womens Health. 2024 Apr 2;24:212. doi: 10.1186/s12905-024-03059-2 (PMC10986057; doi:10.1186/s12905-024-03059-2)
Supplement: Supplementary file 2 — Supplementary Material 2 [file 12905_2024_3059_MOESM2_ESM.docx]

Supplementary Table 1 Characteristics of non-weighted study participants according to SUI, NHANES 2005 to 2018 (n = 10, 137).

| Characteristics | Total  (n = 10, 137) | Non-SUI  (n = 5, 979) | SUI  (n = 4, 158) | *P* value |
| --- | --- | --- | --- | --- |
| Age (years) |  |  |  | < 0.0001 |
| 20-40 | 3946(38.93) | 2811(48.87) | 1135(26.84) |  |
| 41-60 | 3587(35.39) | 1805(32.38) | 1782(46.98) |  |
| ≥61 | 2604(25.69) | 1363(18.76) | 1241(26.18) |  |
| Race |  |  |  | < 0.0001 |
| Non-Hispanic White | 4642(45.79) | 2549(68.64) | 2093(75.39) |  |
| Non-Hispanic Black | 2061(20.33) | 1431(12.53) | 630(7.44) |  |
| Mexican American | 1409(13.9) | 741(6.24) | 668(6.78) |  |
| Others | 2025(19.98) | 1258(12.59) | 767(10.39) |  |
| Education levels |  |  |  | < 0.001 |
| Less than high school | 1819(17.94) | 996(10.50) | 823(12.38) |  |
| High school diploma | 2109(20.8) | 1188(19.28) | 921(22.04) |  |
| More than high school | 6209(61.25) | 3795(70.22) | 2414(65.58) |  |
| Marriage status |  |  |  | < 0.0001 |
| Never married | 1946(19.2) | 1456(22.62) | 490(10.03) |  |
| Separated | 2577(25.42) | 1390(19.57) | 1187(23.70) |  |
| Married | 5614(55.38) | 3133(57.81) | 2481(66.27) |  |
| Family income |  |  |  | 0.108 |
| < $25,000 | 2322(22.91) | 1387(17.04) | 935(14.83) |  |
| $25,000-$54,999 | 3917(38.64) | 2297(34.46) | 1620(34.27) |  |
| $55,000-$99,999 | 2344(23.12) | 1370(27.31) | 974(28.82) |  |
| ≥ $100,000 | 1554(15.33) | 925(21.19) | 629(22.09) |  |
| Family PIR |  |  |  | 0.554 |
| < 1.3 | 3738(36.87) | 2218(34.87) | 1520(34.81) |  |
| 1.3-3.5 | 2987(29.47) | 1742(19.88) | 1245(18.98) |  |
| ≥ 3.5 | 3412(33.66) | 2019(45.25) | 1393(46.21) |  |
| Alcohol drinking status |  |  |  | 0.182 |
| Never | 1841(18.16) | 1106(20.18) | 735(18.31) |  |
| Moderate | 5199(51.29) | 3060(56.30) | 2139(57.65) |  |
| Heavy | 3097(30.55) | 1813(23.52) | 1284(24.03) |  |
| Smoking status |  |  |  | < 0.0001 |
| Never | 6450(63.63) | 3989(64.30) | 2461(57.21) |  |
| Current | 1793(17.69) | 973(16.47) | 820(19.46) |  |
| Former | 1894(18.68) | 1017(19.23) | 877(23.33) |  |
| Physical activity |  |  |  | < 0.001 |
| Low | 2540(25.06) | 1448(22.99) | 1092(25.47) |  |
| Moderate | 1717(16.94) | 983(15.71) | 734(18.48) |  |
| High | 5880(58.01) | 3548(61.31) | 2332(56.05) |  |
| Pregnant history (yes, %) | 8284(81.72) | 4538(71.77) | 3746(88.80) |  |
| Menopause (yes, %) | 4813(47.48) | 2526(40.18) | 2287(53.96) | < 0.0001 |
| Gynecological cancer (yes, %) | 140(1.381) | 59(1.177) | 81(2.284) | 0.002 |
| Diabetes (yes, %) | 1465(14.45) | 708(8.19) | 757(14.15) | < 0.0001 |
| Cardiovascular disease (yes, %) | 683(6.74) | 315(4.11) | 368(7.41) | < 0.0001 |
| BMI, kg/m^2^ | 28.65(0.118) | 27.80(0.135) | 29.80(0.177) | < 0.0001 |
| Waist circumference, cm | 95.32(0.274) | 93.00(0.318) | 98.47(0.393) | < 0.0001 |
| ABSI | 0.080(0.000) | 0.080(0.000) | 0.081(0.000) | < 0.0001 |
| BRI | 5.357(0.040) | 5.017(0.046) | 5.818(0.061) | < 0.0001 |
| CI | 1.286(0.002) | 1.272(0.002) | 1.304(0.002) | < 0.0001 |
| WHtR | 0.587(0.002) | 0.572(0.002) | 0.608(0.002) | < 0.0001 |
| Trunk fat ratio | 0.450(0.002) | 0.440(0.002) | 0.465(0.002) | < 0.0001 |

Data were presented as the mean ± standard error (continuous) or number with percent (categorical). BMI, body mass index; PIR, poverty-income ratio; WHtR, waist-to-height ratio; CI, conicity index; ABSI, a body shape index; BRI, body round index.
