# Supplementary material for: Abdominal obesity as assessed by anthropometric measures associates with urinary incontinence in females: findings from the National Health and Nutrition Examination Survey 2005–2018
Source: BMC Womens Health. 2024 Apr 2;24:212. doi: 10.1186/s12905-024-03059-2 (PMC10986057; doi:10.1186/s12905-024-03059-2)
Supplement: Supplementary file 3 — Supplementary Material 3 [file 12905_2024_3059_MOESM3_ESM.docx]

Supplementary Table 2 Characteristics of non-weighted study participants according to UUI, NHANES 2005 to 2018 (n = 10, 137).

| Characteristics | Total  (n = 10, 137) | Non-UUI  (n = 7, 400) | UUI  (n = 2, 737) | *P* value |
| --- | --- | --- | --- | --- |
| Age (years) |  |  |  | < 0.0001 |
| 20-40 | 3946(38.93) | 3346(45.43) | 600(21.77) |  |
| 41-60 | 3587(35.39) | 2534(37.32) | 1053(42.34) |  |
| ≥61 | 2604(25.69) | 1520(17.25) | 1084(35.89) |  |
| Race |  |  |  | < 0.0001 |
| Non-Hispanic White | 4642(45.79) | 3383(71.40) | 1259(71.84) |  |
| Non-Hispanic Black | 2061(20.33) | 1399(9.66) | 662(12.49) |  |
| Mexican American | 1409(13.9) | 1019(6.50) | 390(6.37) |  |
| Others | 2025(19.98) | 1599(12.44) | 426(9.30) |  |
| Education levels |  |  |  | < 0.0001 |
| Less than high school | 1819(17.94) | 1233(10.32) | 586(14.25) |  |
| High school diploma | 2109(20.8) | 1487(19.67) | 622(22.79) |  |
| More than high school | 6209(61.25) | 4680(70.01) | 1529(62.96) |  |
| Marriage status |  |  |  | < 0.0001 |
| Never married | 1946(19.2) | 1527(18.63) | 419(13.22) |  |
| Separated | 2577(25.42) | 1634(18.75) | 943(29.04) |  |
| Married | 5614(55.38) | 4239(62.62) | 1375(57.74) |  |
| Family income |  |  |  | < 0.0001 |
| < $25,000 | 2322(22.91) | 1590(15.14) | 732(18.99) |  |
| $25,000-$54,999 | 3917(38.64) | 2824(33.80) | 1093(36.12) |  |
| $55,000-$99,999 | 2344(23.12) | 1753(28.19) | 591(27.24) |  |
| ≥ $100,000 | 1554(15.33) | 1233(22.87) | 321(17.66) |  |
| Family PIR |  |  |  | 0.002 |
| < 1.3 | 3738(36.87) | 2706(34.18) | 1032(36.85) |  |
| 1.3-3.5 | 2987(29.47) | 2098(18.83) | 889(21.49) |  |
| ≥ 3.5 | 3412(33.66) | 2596(46.99) | 816(41.66) |  |
| Alcohol drinking status |  |  |  | 0.002 |
| Never | 1841(18.16) | 1394(20.40) | 447(16.35) |  |
| Moderate | 5199(51.29) | 3791(56.46) | 1408(58.11) |  |
| Heavy | 3097(30.55) | 2215(23.14) | 882(25.54) |  |
| Smoking status |  |  |  | < 0.001 |
| Never | 6450(63.63) | 4838(62.75) | 1612(56.90) |  |
| Current | 1793(17.69) | 1261(17.16) | 532(19.49) |  |
| Former | 1894(18.68) | 1301(20.09) | 593(23.61) |  |
| Physical activity |  |  |  | < 0.0001 |
| Low | 2540(25.06) | 1749(22.70) | 791(28.07) |  |
| Moderate | 1717(16.94) | 1258(16.93) | 459(16.75) |  |
| High | 5880(58.01) | 4393(60.37) | 1487(55.18) |  |
| Pregnant history (yes, %) | 8284(81.72) | 5855(76.32) | 2429(87.04) | < 0.0001 |
| Menopause (yes, %) | 4813(47.48) | 3025(40.00) | 1788(64.13) | < 0.0001 |
| Gynecological cancer (yes, %) | 140(1.381) | 81(1.287) | 59(2.726) | < 0.0001 |
| Diabetes (yes, %) | 1465(14.45) | 889(8.79) | 576(16.51) | < 0.0001 |
| Cardiovascular disease (yes, %) | 683(6.74) | 359(3.94) | 324(10.21) | < 0.0001 |
| BMI, kg/m^2^ | 28.650(0.118) | 28.079(0.132) | 30.361(0.181) | < 0.0001 |
| Waist circumference, cm | 95.324(0.274) | 93.790(0.298) | 99.925(0.405) | < 0.0001 |
| ABSI | 0.080(0.000) | 0.080(0.000) | 0.081(0.000) | < 0.0001 |
| BRI | 5.357(0.040) | 5.125(0.044) | 6.052(0.063) | < 0.0001 |
| CI | 1.286(0.002) | 1.277(0.002) | 1.312(0.003) | < 0.0001 |
| WHtR | 0.587(0.002) | 0.577(0.002) | 0.618(0.003) | < 0.0001 |
| Trunk fat ratio | 0.450(0.002) | 0.447(0.002) | 0.460(0.003) | < 0.001 |

Data were presented as the mean ± standard error (continuous) or number with percent (categorical). BMI, body mass index; PIR, poverty-income ratio; WHtR, waist-to-height ratio; CI, conicity index; ABSI, a body shape index; BRI, body round index.
