# Supplementary material for: Abdominal obesity as assessed by anthropometric measures associates with urinary incontinence in females: findings from the National Health and Nutrition Examination Survey 2005–2018
Source: BMC Womens Health. 2024 Apr 2;24:212. doi: 10.1186/s12905-024-03059-2 (PMC10986057; doi:10.1186/s12905-024-03059-2)
Supplement: Supplementary file 4 — Supplementary Material 4 [file 12905_2024_3059_MOESM4_ESM.docx]

Supplementary Table 3 Spearman rank correlation matrix for seven variables representing anthropometric measurements and obesity in 10, 137 female participants.

|  | **BMI** | **BRI** | **CI** | **WHtR** | **ABSI** | **Waist** | **Total fat** | **Trunk fat** | **Trunk fat ratio** |
| --- | --- | --- | --- | --- | --- | --- | --- | --- | --- |
| **BMI** |  |  |  |  |  |  |  |  |  |
| **BRI** | 0.924 |  |  |  |  |  |  |  |  |
| **CI** | 0.502 | 0.759 |  |  |  |  |  |  |  |
| **WHtR** | 0.920 | 0.993 | 0.779 |  |  |  |  |  |  |
| **ABSI** | -0.063 | 0.282 | 0.827 | 0.300 |  |  |  |  |  |
| **Waist** | 0.923 | 0.963 | 0.773 | 0.967 | 0.293 |  |  |  |  |
| **Total fat** | 0.950 | 0.828 | 0.584 | 0.879 | 0.062 | 0.926 |  |  |  |
| **Trunk fat** | 0.935 | 0.917 | 0.685 | 0.920 | 0.192 | 0.953 | 0.966 |  |  |
| **Trunk fat ratio** | 0.481 | 0.604 | 0.686 | 0.637 | 0.486 | 0.589 | 0.445 | 0.641 |  |

All *P* value < 0.001; BMI, body mass index; WHtR, waist-to-height ratio; CI, conicity index; ABSI, a body shape index; BRI, body round index.
